# Relationship between Functional Visual Acuity and Useful Field of View in Elderly Drivers

**DOI:** 10.1371/journal.pone.0147516

**Published:** 2016-01-25

**Authors:** Kazuno Negishi, Sachiko Masui, Masaru Mimura, Yoshio Fujita, Kazuo Tsubota

**Affiliations:** 1 Department of Ophthalmology, Keio University School of Medicine, Tokyo, Japan; 2 Department of Neuropsychiatry, Keio University School of Medicine, Tokyo, Japan; 3 Faculty of Health Sciences, Mejiro University, Tokyo, Japan; University Medical Center Goettingen, GERMANY

## Abstract

**Purpose:**

To investigate the relationship between the functional visual acuity (FVA) and useful field of view (UFOV) in elderly drivers and assess the usefulness of the FVA test to screen driving aptitude in elderly drivers.

**Methods:**

This study included 45 elderly drivers (31 men, 14 women; mean age, 68.1 years) and 30 younger drivers (26 men, 4 women; mean age, 34.2 years) who drive regularly. All participants underwent measurement of the binocular corrected distant visual acuity (CDVA), binocular corrected distant FVA (CDFVA), and Visual Field with Inhibitory Tasks Elderly Version (VFIT-EV) to measure UFOV. The tear function and cognitive status also were evaluated.

**Results:**

The CDVA, the CDFVA, cognitive status, and the correct response rate (CAR) of the VFIT-EV were significantly worse in the elderly group than in the control group (*P* = 0.000 for all parameters). The cognitive status was correlated significantly with the CDVA (r = -0.301, *P* = 0.009), CDFVA (r = -0.402, *P* = 0.000), and the CAR of the VFIT-EV (r = 0.348, *P* = 0.002) in all subjects. The results of the tear function tests were not correlated with the CDVA, CDFVA, or VFIT-EV in any subjects. Stepwise regression analysis for all subjects in the elderly and control groups showed that the CDFVA predicted the CAR most significantly among the clinical factors evaluated.

**Conclusion:**

The FVA test is a promising method to screen the driving aptitude, including both visual and cognitive functions, in a short time.

## Introduction

Rapid growth in the number of elderly individuals and the proportion of the population that is aged in North America, Europe, Asia, and Australia is expected in the near future [1], raising concerns about the safety of elderly drivers. Nonetheless, more appropriate techniques to perform screenings for proper driving aptitude with the goal of preventing accidents caused by elderly drivers are still under debate worldwide.

Vision, cognition, and motor functions are three key domains required for safe driving [2], and researchers agree that vision plays a significant role in driving performance. A number of visual function tests are used for drivers to qualify for obtaining a driver’s license, and the tests vary from country to country, including measurement of visual acuity (VA), contrast sensitivity, and depth perception [3]; however, the effectiveness of the test for evaluating the driving aptitude remains unclear [1, 3]. There is a need to develop evidence-based and validated tools for vision screening to ensure driving safety.

The functional visual acuity (FVA) recently has been reported to be an important method for measuring the detailed visual performance that cannot be detected by conventional VA testing [4–8]. The definition of FVA has been suggested to indicate the visual performance related to certain daily activities such as driving, reading, and use of a visual display terminal [8]. However, the effectiveness of FVA testing for vision screening to ensure safe driving has not been validated.

The cognitive ability, i.e., the useful field of view (UFOV), is considered a good predictor of at-fault crash risk in elderly drivers [9–11]. The UFOV originally was defined as a measure of task performance, representing the visual area over which necessary information can be acquired in a brief glance without eye or head movement [12]. The UFOV has been reported to decline with age [9, 12–14].

The conventional UFOV test consists of dual-task testing with simultaneous central and peripheral targets. The test requires a touch panel screen or other complicated device and is subject to bodily and language functions [9]. However, several modified methods to improve these weaknesses have been reported recently. Fujita et al. developed a new UFOV test, i.e., the visual field with inhibitory tasks (VFIT) and its version for screening elderly individuals (VFIT-EV), to resolve these problems [15]. The authors reported that the results of the VFIT-EV were correlated significantly with the results of an actual vehicular test for Japanese driver’s license renewal by elderly drivers and suggested that the VFIT may be a good predictor of the driving aptitude [15].

We investigated the relationship between the results of the FVA test and the VFIT-EV in elderly and young drivers and the possibility of using the FVA test to screen the driving aptitude of elderly drivers.

## Methods

### Participants and Test Conditions

This prospective study included 45 elderly individuals (elderly group) (age 60 years or older) who drove regularly. Thirty younger individuals (control group) (age 59 years or younger) who drove regularly were also included. The study inclusion criteria were general good health, driving frequency greater than once weekly, and possession of a current driver’s license. In Japan, a binocular VA of 0.7 (20/29) or better and a bilateral monocular VA of 0.3 (20/60) or better are required and tested at 5-year intervals at the driver license center during the process of driver’s license renewal. The exclusion criterion was the presence of systemic or ocular diseases that might affect driving.

The elderly group included 31 men and 14 women. The mean age of the participants was 68.1 ± 4.4 years (standard deviation) (range, 60–77 years). The control group included 26 men and four women. The mean age of the participants was 34.2 ± 7.9 years range, 22–51 years). All examinations were performed in a room in which the temperature was between 22 and 25 degrees centigrade, and the mean humidity was 27.0% ± 4.8%.

### Ethics Statement

The institutional review board of Keio University School of Medicine approved the study (approval number: 20120324). All subjects provided written informed consent. The study adhered to the tenets of the Declaration of Helsinki. The individuals who participated in the study provided written informed consent to publish the case details.

### Visual Function Tests

**Fig 1:**
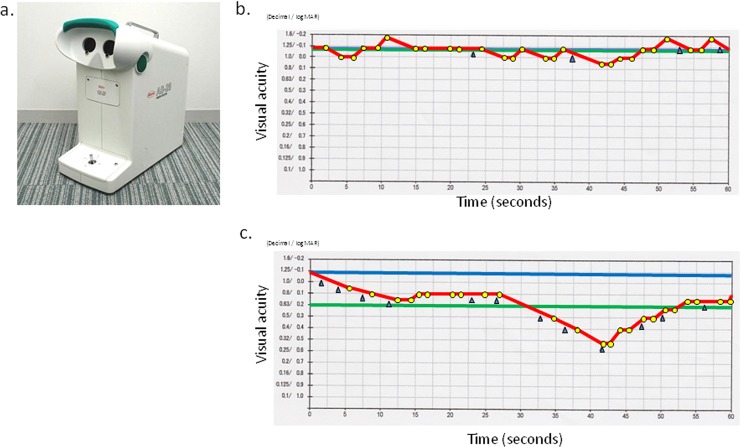
FVA Measurement System and results of typical cases. **(A)** FVA Measurement System (AS-28, Kowa). **(B)** Representative printout of a typical case with good FVA. The blue line denotes the Landolt corrected VA. The red line shows the time-wise changes in the VA during testing. The green line denotes the mean logMAR VA during 60 seconds, defined as the FVA. The yellow dots indicate the number of correct responses and the blue arrows indicate spontaneous blinks. FVA (logMAR), -0.08; VMR, 0.99; max/min logMAR VAs, -0.18/0.05, respectively; ART, 1.47 seconds. **(C)** Representative printout of a typical case with low FVA. FVA (logMAR), 0.19; VMR, 0.83; max/min logMAR VA, 0.05/0.52, respectively; ART, 1.46 seconds.

Binocular measures were used for visual function tests because driving is typically performed with both eyes. All participants underwent measurement of the binocular corrected distant VA (binocular CDVA) using Landolt vision charts and the binocular corrected distant functional VA (CDFVA) using the AS-28 FVA Measurement System (Kowa, Aichi, Japan) (Fig 1A), which was used to examine the changes in continuous VA over 60 seconds. The Landolt optotypes were presented in the device for 2 seconds, and their changes in size depended on the correctness of the responses. The measurement started with the best-corrected Landolt VA, which was the baseline VA of each individual. The Landolt optotypes decreased in size automatically when a correct response was given. If a response was incorrect, a larger optotype was presented automatically. When no response was forthcoming within the set display time, this was considered erroneous and the optotype enlarged automatically. Subjects delineated the orientation of the automatically presented Landolt rings from the baseline VA using a joystick. The system can measure VA levels from 20/13 to 20/200. The testing was performed with spontaneous blinking. The continuous VA changes were plotted in yellow (Fig 1B). The FVA measurement included several evaluation indices: the FVA, maximal and minimal VA (maxVA and minVA), visual maintenance ratio (VMR), and average response time (ART). The FVA was defined as the average of all VA values measured over time. The VMR was calculated as follows: VMR = (lowest logarithm of the minimum angle of resolution [logMAR] VA score—FVA at 60 seconds)/(lowest logMAR VA score—baseline VA) [4, 16]. The FVA was measured with the best distance correction. The subjects practiced for 60 seconds before the measurement to eliminate the effect of unfamiliarity with the test.

### Tear Function Evaluation and Cognitive Status

The tear function was evaluated in all subjects because it affects visual function, especially the FVA [17, 18]. The standard Schirmer’s test without topical anesthesia and the standard tear break-up time (BUT) measurement were performed for all subjects. Standardized strips of filter paper (Showa Yakuhin, Tokyo, Japan) were placed in the lateral canthus away from the cornea and left in place for 5 minutes with the eyes closed. The BUT was measured after instillation of fluorescein sodium in the conjunctival sac with a test filter paper. The interval between the last complete blink and the appearance of the first corneal black spot in the stained tear film was measured three times, and the mean value of the measurements was calculated.

The Mini-Mental State Examination–Japanese (MMSE-J, Nihon Bunka Kagakusha Co., Ltd.) was performed to evaluate cognitive status.

### Self-Reporting Questionnaire

Data on the years of driving experience, kilometers driven annually, and the self-reported number of motor vehicle collisions (MVCs) during the previous 5 years were collected through a self-reporting questionnaire for all participants.

### UFOV

**Fig 2:**
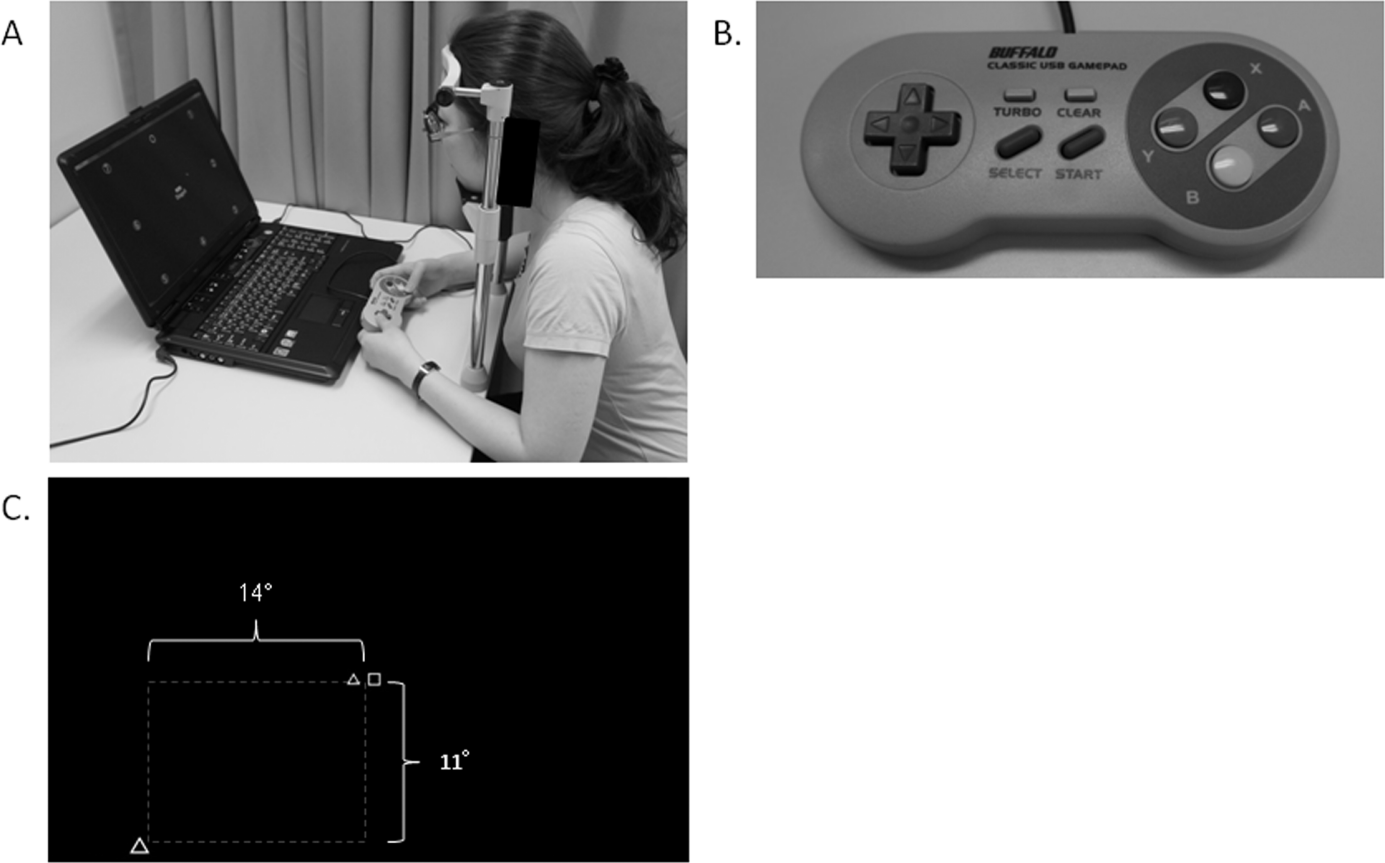
Useful field of view with the VFIT-EV test. **(A)** Useful field of view with the VFIT-EV test. **(B)** Game controller used to respond during the VFIT-EV test. **(C)** Screen display of the VFIT-EV, dual-task test (actual test).

The UFOV was measured using a visual field with the VFIT-EV (Fig 2A), proposed by Fujita et al. [15]. Stimulus presentation was performed using a monitor on a personal computer (Windows XP, Microsoft on FMVNV60L/W, Fujitsu Limited, Tokyo, Japan) with a game controller (BSGP801GY, Buffalo Inc., Aichi, Japan) (Fig 2B). Participants sat in front of the screen with their chin on a board to fix the distance between the face and the screen at 50 cm. The VFIT-EV was measured with the best correction at 50 cm. The examiner confirmed that the participants could use the game controller without looking at it directly before the trial. During the test, the participants held the game controller in front of their chest or put it on the desk.

The VFIT-EV consists of the three preliminary tests and an actual test. The three preliminary tests were the simple reaction test, the central discrimination test, and the peripheral field test. The actual test is the dual-task test.

The VFIT-EV was conducted as follows. First, the examiner displayed a cross in the center of the screen to confirm the central fixation point for the participant.

#### Simple Reaction Test (Preliminary Test)

After presentation of the central fixation cross for 500 ms, two identical adjoining targets were chosen randomly from among an open circle, open triangle, open square, and open star and presented simultaneously in the center of the monitor for 200 ms. The participants were instructed to push the A button on the right side of the controller when the targets were presented. The software automatically recorded the participant’s response as no response when the participant did not push the button within 2,000 ms and automatically moved to the next stimulus. The participants carried out only one trial in a run.

#### Central Discrimination Test (Preliminary Test)

After presentation of the central fixation cross for 500 ms, two adjoining targets were chosen randomly from among an open circle, open triangle, open square, and open star and presented simultaneously in the center of the monitor for 200 ms. The participants were instructed to push the A button on the right side of the controller when the two central targets differed and to not push the button when the two targets were the same. The software automatically recorded the participant’s response as no response when the participant did not push the button within 2,000 ms and automatically moved to the next stimulus. The participants carried out 32 trials in a run.

#### Peripheral Field Test (Preliminary Test)

After presentation of the central fixation cross for 500 ms, a peripheral target chosen randomly from among the open circle, open triangle, open square and, open star was presented for 200 ms at the peripheral locus randomly chosen from the eight locations: top, top right, right, bottom right, bottom, bottom left, left, and top left. The peripheral targets were at 11 degrees of the visual angle positioned at the top and bottom, at 14 degrees of the visual angle positioned at the right and left, and at the square root degree of the sum of the squares of 11 and 14 at the top right, bottom right, bottom left, and top left, respectively. After the 2,000-ms interval, the figures were presented in the center of the monitor, and the participants were instructed to select the same figure as presented in the periphery using the directional key on the game controller. The software automatically recorded the participant’s response as no response when the participant did not push the button within 2,000 ms and automatically moved to the next stimulus. The participants carried out 16 trials in a run.

#### Dual-Task Test (Actual Test)

After presentation of the central fixation cross for 500 ms, two central targets and one peripheral target were simultaneously presented for 200 ms. The peripheral targets were at 11 degrees of the visual angle positioned at the top and bottom, at 14 degrees of the visual angle positioned at the right and left, and at the square root degree of the sum of the squares of 11 and 14 at the top right, bottom right, bottom left, and top left, respectively (Fig 2C). The interval between the disappearance of the central fixation cross and the presentation of the targets was chosen randomly from 1,500 to 2,900 ms. The participants were instructed to push the A button on the right side of the controller when the two central targets differed and to not push the button when the two targets were the same. After pushing the A button, the figures were presented in the center of the monitor, and the participant was instructed to select the same figure as presented in the periphery using a directional key on the controller. The response was recorded as correct only when the response for both the central figures and the peripheral target were correct. The software automatically recorded the participant’s response as no response when the participant did not push the button within 2,000 ms and automatically moved to the next stimulus. No feedback regarding responses was provided. After the 16 exercise trials performed to reduce mistakes caused by unfamiliarity with the test, the participants carried out 64 trials in a run, among which the same figures were presented in 32 trials. The correct answer rate (CAR) was defined by the rate of the correct answers for 32 trials in which the center figures differed in a run. The false alarm rate (FAR) was defined as the rate of wrong responses for the central targets, i.e., pushing the button when the two central targets were the same.

### Statistical Analysis

The CDVA and CDFVA were compared using the Wilcoxon matched-pairs signed-ranks test. The Spearman’s rank correlation coefficient was used to analyze the correlations between the binocular CDFVA and the CAR of the VFIT-EV. Multivariate analysis with stepwise regression was performed to investigate the relation between the CAR of the VFIT-EV and clinical parameters including age, sex, self-reported number of MVCs during the past 5 years, refraction (average spherical equivalent in both eyes), average values of the tear function tests (BUT and Schirmer’s tests) in both eyes, MMSE, and the parameters of the bilateral FVA (CDFVA, maxVA, minVA, VMR, and ART) and CDVA. *P* < 0.05 was considered statistically significant. All statistical analyses were performed using SPSS software version 12.0J for Windows (IBM, Armonk, NY).

## Results

### Visual Function Tests

Table 1 shows the refraction and VAs of the participants.

**Table 1:** Refraction and visual function.

	Elderly group	Control group	
Parameter	Mean ± SD (range)	Mean ± SD (range)	*P* value
**Spherical equivalent (diopters)**	**-0.46 ± 2.55 (-7.88 ~ 3.50)**	**-4.05 ± 3.55 (-12.75 ~ 0.00)**	**.000**
**CDVA**	**-0.117 ± 0.108 (-0.301 ~ 0.261)**	**-0.201 ± 0.077 (-0.301 ~ 0.000)**	**.000**
**CDFVA**	**-0.031 ± 0.128 (-0.180 ~ 0.538)**	**-0.135 ± 0.079 (-0.180 ~ 0.250)**	**.000**
**MaxVA**	**-0.127 ± 0.110 (-0.180 ~ 0.400)**	**-0.167 ± 0.054 (-0.180 ~ 0.100)**	**.004**
**MinVA**	**0.085 ± 0.170 (-0.18 ~ 0.850)**	**-0.034 ± 0.102 (-0.180 ~ 0.400)**	**.000**
**VMR**	**0.97 ± 0.03 (0.89 ~ 1.03)**	**0.98 ± 0.04 (0.83 ~ 1.05)**	**.070**
**ART (seconds)**	**1.32 ± 0.13 (1.03 ~ 1.61)**	**1.22 ± 0.13 (1.00 ~ 1.47)**	**.003**

The CDVA and all parameters of the FVA test except for the VMR were significantly better in the control group than in the elderly group. The binocular CDVA was significantly higher than the binocular CDFVA in both of the control and the elderly groups (*P =* 0.000, respectively).

### Tear Function Evaluation and Cognitive Status

The mean values of the Schirmer’s test and BUT were 8.6 ± 7.4 (range, 0~35) mm and 4.1± 1.4 seconds (range, 1~14) in the elderly group, and 10.8 ± 10.3 (range, 1~35) mm and 5.0± 2.6 seconds (range, 1~12) in the control group, respectively. There was no significant (*P* = 0.638) difference in the value of the Schirmer’s test, but the value of the BUT was significantly (*P* = 0.033) shorter in the elderly group than in the control group. All cases in the both groups did not fulfill the new Japanese dry eye diagnosis criteria [19]. The mean MMSE-J score was 27.3 ± 2.0 (range, 23–30) in the elderly group and 29.7 ± 0.6 (range, 27–30) in the control group. The MMSE-J score was significantly lower in the elderly group than in the control group (*P* = 0.000).

### Self-Reporting Questionnaire

The mean values of the years of driving experience and the kilometers driven annually were 39.6 ± 12.5 (SD) (range, 10 ~ 58) years and 396.4 ± 308.6 (SD) (range, 30 ~ 1,200) kilometers in the elderly group and 14.2 ± 8.6 (range, 2 ~ 30) years and 1717.6 ± 1757.0 (range, 80 ~ 10,000) in the control group, respectively. The values of the years of driving experience and the kilometers driven annually differed significantly between the groups (*P* = 0.000 for both comparisons). Six of 45 subjects in the elderly group and six of 30 subjects in the control group reported only one motor vehicle collision during the previous 5 years irrespective of the accident severity; however, the rate did not differ significantly between the groups (*P* = 0.526).

### UFOV

Table 2 shows the results of the VFIT-EV for all participants. The CAR was significantly worse in the elderly group than in the control group.

**Table 2:** Results of the VFIT-EV.

	Elderly group	Control group	
Parameter	Mean ± SD (range)	Mean ± SD (range)	*P* value
**CAR (%)**	**84.9 ± 12.1 (53.0 ~ 100.0)**	**96.6 ± 3.9 (87.0 ~ 100.0)**	**.000**
**FAR (%)**	**2.6 ± 3.6 (0 ~ 12.0)**	**2.9 ± 3.9 (0 ~ 15.0)**	**.812**

### Correlations between the FVA and the VFIT-EV Parameters

The correlations between the CDVA and the parameters of the FVA and VFIT-EV in the elderly group are shown in Table 3. The binocular CDVA was correlated significantly with the binocular CDFVA (r = 0.572, *P* = 0.000), maxVA (r = 0.627, *P* = 0.000), and minVA (r = 0.545, *P* = 0.000). The binocular CDFVA was correlated significantly with the binocular maxVA (r = 0.767, *P* = 0.000), minVA (r = 0.952, *P* = 0.000), VMR (r = -0.762, *P* = 0.000), and ART (r = 0.465, *P* = 0.000). The VFIT-EV parameters, the CAR and the FAR, were not correlated with each other (*P* > 0.05). The binocular CDFVA was correlated significantly with the CAR of the VFIT-EV (r = -0.606, *P* = 0.000), although the CDVA was not correlated with the CAR of the VFIT-EV (r = -0.282, *P* = 0.061).

**Table 3:** Spearman Rank Correlation of the CDVA, Parameters of the CDFVA, and the VFIT-EV with Each Other (n = 45)^a^.

**CDVA**							
***r = 0*.*572***	**CDFVA**						
***P = 0*.*000***							
***r = 0*.*627***	***r = 0*.*767***	**maxVA**					
***P = 0*.*000***	***P = 0*.*000***						
***r = 0*.*545***	***r = 0*.*952***	***r = 0*.*723***	**minVA**				
***P = 0*.*000***	***P = 0*.*000***	***P = 0*.*000***					
**r = -0.002**	***r = -0*.*762***	***r = -0*.*485***	***r = -0*.*729***	**VMR**			
**P = -0.989**	***P = 0*.*000***	***P = 0*.*001***	***P = 0*.*000***				
**r = 0.280**	***r = 0*.*465***	**r = 0.199**	***r = 0*.*423***	***r = -0*.*325***	**ART**		
**P = -0.062**	***P = 0*.*001***	**P = 0.190**	***P = 0*.*004***	***P = 0*.*029***			
**r = -0.282**	***r = -0*.*606***	***r = -0*.*356***	***r = -0*.*551***	***r = 0*.*516***	**r = - 0.279**	**CAR**	
**P = 0.061**	***P = 0*.*000***	***P = 0*.*017***	***P = 0*.*000***	***P = 0*.*000***	**P = 0.064**		
**r = 0.018**	**r = 0.110**	**r = 0.038**	**r = 0.102**	**r = -0.137**	**r = 0.230**	**r = - 0.185**	**FAR**
**P = 0.909**	**P = 0.473**	**P = 0.080**	**P = 0.0506**	**P = 0.371**	**P = 0.128**	**P = 0.223**	

^a^Spearman rank correlation coefficients (r values) are shown with the statistical significance of the correlation. Significant correlations are in Italic.

The correlations between the CDVA and the parameters of the FVA and VFIT-EV in the control group are shown in Table 4. The binocular CDVA was correlated significantly with the binocular CDFVA (r = 0.523, *P* = 0.003), minVA (r = 0.420, *P* = 0.021), ART (r = 0.387, *P* = 0.035), and FAR (r = 0.521, *P* = 0.003). The binocular CDFVA was correlated significantly with the binocular minVA (r = 0.746, *P* = 0.000) and VMR (r = -0.754, *P* = 0.000). The VFIT-EV parameters, the CAR and the FAR, were correlated with each other (r = -0.527, *P* = 0.003). Neither the binocular CDVA nor the CDFVA was correlated significantly with the CAR of the VFIT-EV (*P* > 0.05).

**Table 4:** Spearman rank correlation of the CDVA, parameters of the CDFVA, and the VFIT-EV with each other in the control group (n = 30)*.

**CDVA**							
***r = 0*.*523***	**CDFVA**						
***P = 0*.*003***							
**r = 0.317**	**r = 0.211**	**maxVA**					
**P = 0.088**	***P* = 0.263**						
***r = 0*.*420***	***r = 0*.*746***	**r = 0.163**	**minVA**				
***P = 0*.*021***	***P = 0*.*000***	***P* = 0.390**					
**r = -0.170**	***r = -0*.*754***	**r = -0.221**	***r = -0*.*559***	**VMR**			
**P = 0.370**	***P = 0*.*000***	***P* = 0.241**	***P = 0*.*001***				
***r = 0*.*387***	**r = 0.263**	**r = 0.187**	**r = 0.037**	**r = -0.103**	**ART**		
***P = 0*.*035***	**P = 0.161**	***P* = 0.323**	**P = 0.848**	**P = 0.587**			
**r = 0.189**	**r = -0.182**	**r = -0.283**	**r = -0.105**	**r = 0.313**	**r = 0.101**	**CAR**	
**P = 0.317**	**P = 0.337**	**P = 0.129**	**P = 0.582**	**P = 0.092**	**P = 0.595**		
***r = -0*.*521***	**r = -0.032**	**r = -0.250**	**r = -0.063**	**r = -0.055**	**r = -0.160**	***r = -0*.*527***	**FAR**
***P* = 0.003**	***P* = 0.866**	***P* = 0.183**	***P* = 0.742**	***P* = 0.773**	***P* = 0.398**	***P = 0*.*003***	

*Spearman rank correlation coefficients (r values) are shown with the statistical significance of the correlation. Significant correlations are in Italic.

### Correlations between the Visual Function, VFIT-EV, MMSE, and Tear Function Tests

The MMSE was correlated significantly with the CDVA (r = -0.301, *P* = 0.009), CDFVA (r = -0.402, *P* = 0.000), and the CAR of the VFIT-EV (r = 0.348, *P* = 0.002) for all subjects in the elderly and the control groups. The results of the tear function tests were not correlated with the CDVA, CDFVA, or VFIT-EV for all subjects in the elderly and the control groups.

### Stepwise Regression Analysis

The results of stepwise regression analysis in all subjects are shown in Table 5. Stepwise regression analysis with forward selection of predictors showed that the CDFVA, MMSE, and maxVA were significant (r = 0.806, F = 33.285, *P* = 0.000) predictors of the CAR of the VFIT-EV.

**Table 5:** Results of multivariate regression analysis with stepwise selection in terms of the CAR of the VFIT-EV.

Variable	Regression coefficient	Standardized regression coefficient	*P* value	VIF
**CDFVA**	**-89.535**	**-0.822**	**0.000**	**3.392**
**MMSE**	**2.521**	**0.432**	**0.000**	**1.286**
**maxVA**	**66.974**	**0.444**	**0.003**	**3.085**

The multiple regression equation was: CAR of the VFIT-EV = 20.664 + (-89.535 × CDFVA) + (2.521× MMSE) + (66.974 × maxVA).

**Fig 3:**
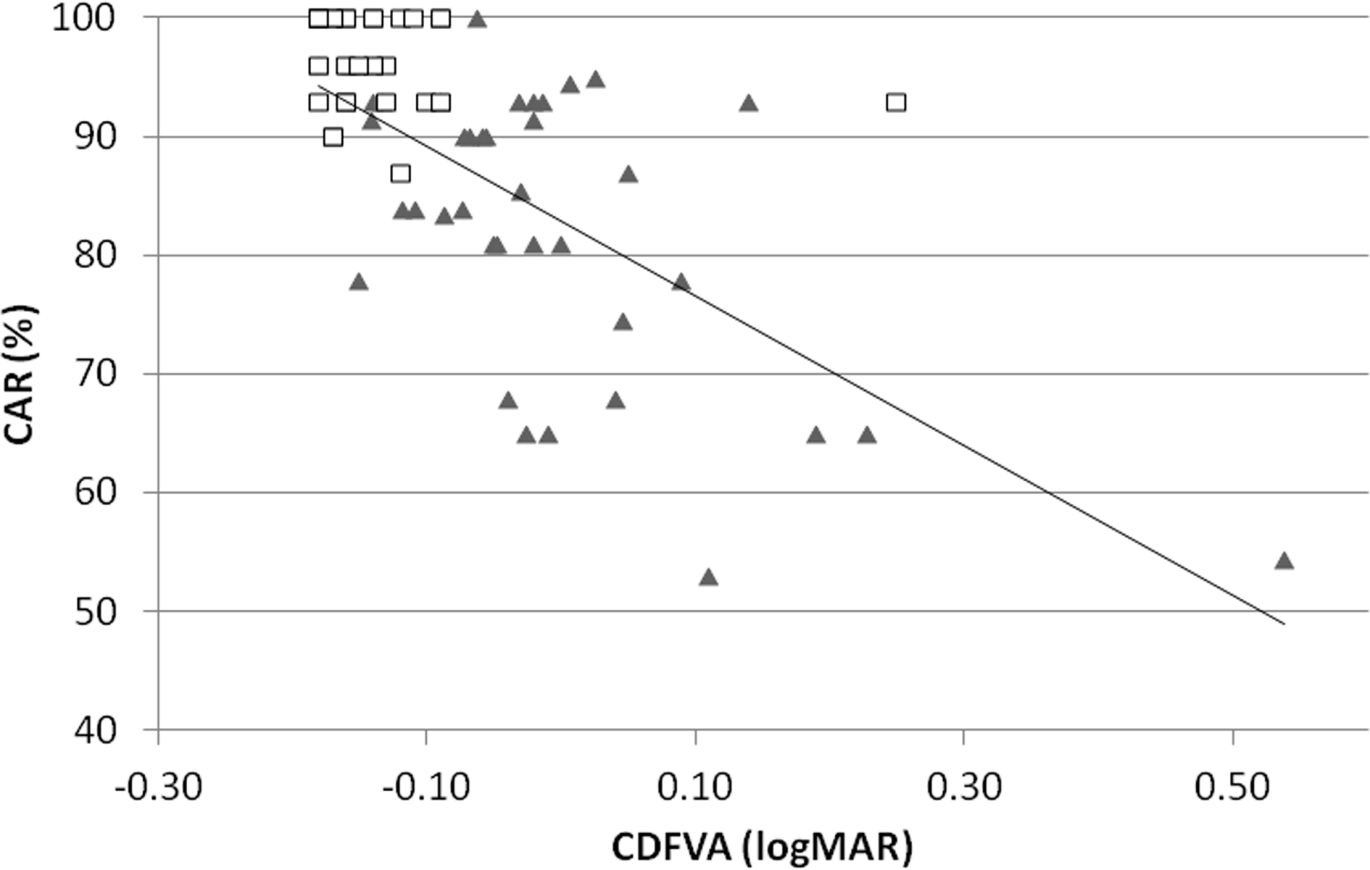
Correlation between the CDFVA and the CAR of the VFIT-EV. The correlation between the CDFVA and the CAR of the VFIT-EV is significant (r = -0.606, *P* = 0.000). The triangles indicate the data from the elderly group, and the squares indicate the data from the control group.

Standardized regression coefficients were assessed to discern the magnitude of the effect of each variable. The CDFVA was the most relevant variable followed by the maxVA (Table 5). The correlation between the CDFVA and the CAR of the VFIT-EV is shown in Fig 3. As shown in the graph, both the CDFVA and the CAR of the VFIT-EV were excellent and had small variations in the control group.

## Discussion

In the current study, the CDFVA in the elderly group was correlated not only with the CDVA but also with the VFIT-EV, which is one of the modified UFOV tests for elderly drivers, although there were no such correlations in the control group. Stepwise regression analysis for all subjects also showed that the CDFVA was the most relevant factor predictive of the CAR of the VFIT-EV, which was correlated significantly with the results of the actual vehicular test of Japanese driver license renewal for elderly drivers followed by the maxVA. The result suggested that the CDFVA may be useful for simultaneous screening of the visual and cognitive functions related to the driving aptitude of elderly drivers.

There are a variety of vision screening methods, and the conventional VA test is the most popular method used by the licensing authorities in many countries, although other visual function tests also are screening tools in some countries [3]. However, it is unclear if the other current vision tests used worldwide can assess the visual functions required to drive safely [3].

The UFOV can examine the minimal target duration required to perform the central discrimination task, the ability to divide attention between central and peripheral tasks, and the ability to filter out distracting stimuli, i.e., the visual processing speed and visual attention can be assessed with the UFOV test [12, 20]. Previous studies have reported a correlation between the UFOV and involvement in MVCs [11, 12, 21]. Owsley and McGwin reported that elderly drivers with 40% or greater impairment of the UFOV were 2.2 times more likely to have a crash during 3 years of follow-up, after adjusting for age, sex, race, chronic medical conditions, mental status, and days driven/week [11]. Cross et al. reported a strong association between the UFOV and MVCs, although there was no significant association between any of the ocular diseases studied (cataract, glaucoma, macular degeneration, diabetic retinopathy) and MVC involvement rates [20]. However, the relationship between MVC involvement and the VFIT-EV is unknown because access to the MVC data is limited to the public security authorities and the police department and the data remain undisclosed in Japan.

However, the CAR of the VFIT-EV is correlated with the results of the on-road actual vehicular test of Japanese driver’s license renewal for elderly drivers [15]. The current results showed that the CDFVA and not the CDVA was correlated significantly with the CAR of the VFIT-EV in the elderly group. Moreover, the results of stepwise regression for all subjects in the elderly and the control groups showed that the CDFVA was the most relevant predictor of the CAR of the VFIT-EV, followed by the maxVA. Recently, the FVA test has been reported to be useful for evaluating degradation of visual function that cannot be detected by conventional VA testing. A previous study reported that the FVA can detect a slight decrease in visual function due to early cataract [22], posterior capsule opacification [23], and astigmatism [7] even in patients with 20/20 and better vision. In addition, and this differs from other visual function tests, the FVA test may include cognitive and motor functions in addition to visual function, because the subjects must answer by moving a joystick immediately after recognizing the Landolt chart. If the subject fails to respond within the time limit, it is considered to be an erroneous answer. This procedure may resemble the central discrimination task in the UFOV. In other words, the FVA test can evaluate visual function and cognitive function partially by presenting the charts using a method similar to the UFOV, which may suggest that the FVA may better fit the visual function test to screen the driving aptitude compared with other visual function tests.

The current study had some limitations, i.e., a small number of participants, differences in the years of driving experience and the kilometers driven annually between the groups, and the lack of data on the actual MVC involvement rate in Japan. According to the results of the questionnaire used in the current study, six of 45 participants in the elderly group and six of the 30 participants in the control group reported that they had a MVC only once during the past 5 years, although the severity of the accidents was unknown. Further investigations are needed to consider these factors.

Another potential limitation is that, different from the UFOV, the FVA test evaluates only the central visual function, although the results of both tests are significantly correlated. Tafaj et al. recently reported a new method that goes beyond the typical assessment of the visual functioning [24]. The method measures the visual exploration capability of a subject in order to understand the actual impact of visual field defects on activities of daily living and potential compensatory strategies. Considering this, the FVA should be improved to evaluate the visual function in a condition closer to reality.

In summary, the FVA test may be a promising method to screen the driving aptitude including both visual and cognitive functions within a short measurement time (60 seconds).
